# Comparison of three DNA extraction methods for recovery of microbial DNA from Arctic permafrost

**DOI:** 10.17912/micropub.biology.000834

**Published:** 2023-06-21

**Authors:** Sarah Feng, Marla DeKlotz, Neslihan Taş

**Affiliations:** 1 Lawrence Berkeley National Laboratory, Berkeley, California, United States

## Abstract

Permafrost soils, which contain one of Earth’s largest terrestrial carbon stocks, are vulnerable to thaw and microbial decomposition, exacerbating climate change. Advancements in sequencing technologies have facilitated the identification and functional profiling of microbial communities in permafrost, but DNA extraction from these soils is challenging due to their high microbial diversity and low biomass. This study assessed the effectiveness of the DNeasy PowerSoil Pro kit in extracting DNA from permafrost samples and found that it produced significantly different results than the discontinued DNeasy PowerSoil kit. The study highlights the importance of consistent DNA extraction methods in permafrost studies.

**Figure 1. Comparison of three DNA extraction kits on DNA quantity and community composition:**
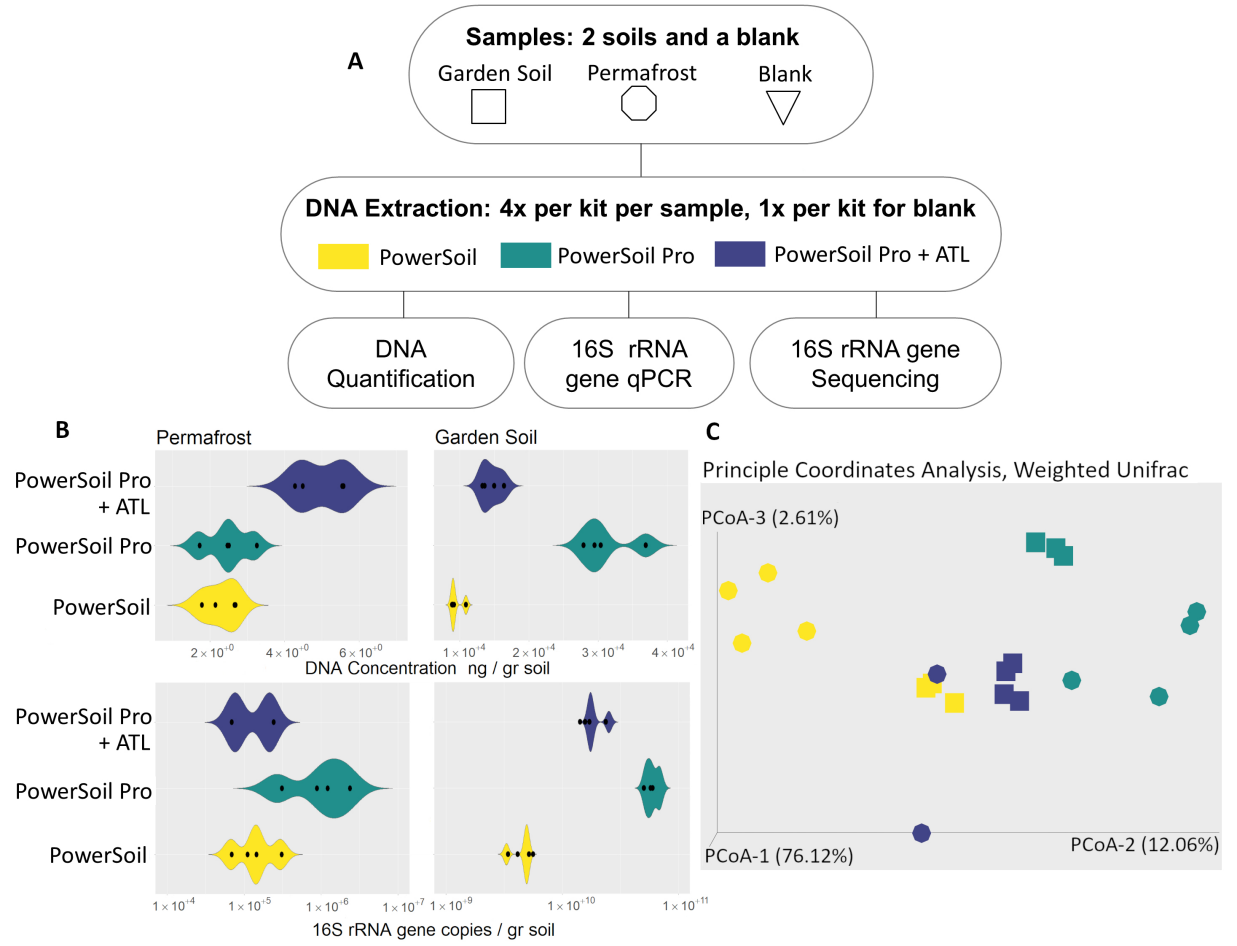
(A) DNA extraction workflow. Permafrost and garden soils were extracted in quadruplicate using DNeasy PowerSoil, DNeasy PowerSoil Pro, and ATL buffer added to DNeasy PowerSoil Pro. Colors correspond to DNA extraction methods and shapes correspond to samples. (B) Extracted DNA concentrations and 16S rRNA gene copies per gram soil were determined by Qubit fluorometric quantification and quantitative PCR (qPCR). One garden soil from the PowerSoil Pro set was lost due to technical error, and two permafrost samples from the DNeasy PowerSoil Pro kit + ATL buffer set did not pass the quality filtering step during the sequencing workflow; these samples were thus dropped from ensuing analyses. (C) Principal coordinate analysis (PCoA) of weighted unifrac distances show sample clustering among different DNA extraction methods.

## Description

Permafrost is defined as ground that remains at or below 0°C for at least two consecutive years (Biskaborn et al., 2019) . Soils in permafrost zones are characterized by high volumes of organic matter, making them one of the largest terrestrial carbon stocks on Earth (Zimov et al., 2006) . As global temperatures continue to rise, permafrost will thaw, creating new habitats that can support increased microbial growth and decomposition rates. This may lead to elevated greenhouse gas emissions and further exacerbate the problem of climate change (Schuur et al., 2013) . In addition, permafrost microbes are uniquely adapted to cold temperatures and able to survive in a state of low metabolic activity (D’amico et al., 2006). Given the crucial role that permafrost microbes play in biogeochemical cycling, it is imperative to gain a better understanding of their functional potential and responses to environmental change. This knowledge may inform strategies for mitigating the impacts of climate change.

Advancements in sequencing technologies have facilitated the identification and functional profiling of microbial communities in permafrost. These technologies require the extraction of high-quality DNA that can be used in analysis methods such as PCR (polymerase chain reaction), as well as be processed for sequencing. Achieving high-quality, representative extracts can be particularly challenging as permafrost soils have high microbial diversity despite having low microbial biomass in comparison to soils from other environments (Jansson and Tas, 2014) . Previous research has shown that among the different commercially available DNA extraction kits, the DNeasy PowerSoil kit has consistently produced high-quality DNA from permafrost samples, with minimal variation among replicates (Vishnivetskaya et al., 2014a). Recently, this kit was discontinued and replaced with the DNeasy PowerSoil Pro kit. Different DNA extraction kits and methods can produce varying results from the same sample, making it crucial to evaluate the impact of extraction chemistry on DNA yield and subsequent analysis of microbial community composition and structure by amplicon sequencing (Vishnivetskaya et al., 2014b). This project aims to address this issue by assessing the efficiency of the DNeasy PowerSoil Pro kit in extracting and identifying microbial DNA from permafrost samples compared to the previously available DNeasy PowerSoil kit.

In this experiment, we used the DNeasy PowerSoil kit, DNeasy PowerSoil Pro kit, and a modified version of the DNeasy PowerSoil Pro kit with Qiagen Buffer ATL on two soil types, a permafrost sample from Anaktuvuk, AK, and a store-bought garden soil. The addition of Qiagen ATL buffer has been proposed to improve cell lysis. We performed four replicates of each extraction method and one water blank as a negative control per soil type, for a total of 30 extractions ( Figure 1A ). Garden soil was used as a reference to compare the DNA yield and quality of the permafrost samples. We used Qubit 3.0 fluorometric quantification to measure concentration of each DNA extraction and 16S rRNA gene-targeted quantitative PCR (qPCR) to assess the abundance of microbial DNA in each sample. 16S libraries were prepared for all samples using the Zymo Quick-16S Plus NGS Library Kit, with the quantification of total 16S copy number per sample measured by real-time PCR. Amplicons from the V3-V4 region of 16S were sequenced to identify changes in microbial community structure originating from the DNA extraction method. Lastly, we used Analysis of Variance (ANOVA) and principal coordinate analysis (PCoA) to determine statistically significant differences in DNA quantity, sequence similarity, and community structure.

We found that different DNA extraction methods yielded varying results. We first compared total DNA yield in each soil type by each extraction method ( Figure 1B ). In permafrost samples, we extracted an average of 3.25 ng DNA per gram of soil, with a range of 1.69 to 5.64 ng DNA per gram of soil. Average extracted DNA yield was not significantly different between the DNeasy PowerSoil Pro and DNeasy PowerSoil extraction kits. The addition of Qiagen ATL buffer to the DNeasy PowerSoil Pro kit resulted in significantly higher DNA from permafrost samples compared to the PowerSoil and PowerSoil Pro kits (n = 12, Welch's F _(2,5.79)_ = 22.40, p = .002). In garden soil samples, DNA yield varied between 8.92 x 10 ^3^ and 3.69 x 10 ^4^ ng DNA per gram of soil, with the PowerSoil Pro kit resulting in the highest DNA extraction yield (n = 12, Welch's F _(2,5.20)_ = 59.95, p < .001).

After conducting 16S rRNA gene-targeted qPCR, we observed variability in the quantities of 16S rRNA gene copies per gram of soil among the kits. While the addition of ATL buffer to the PowerSoil Pro kit increased DNA yield in permafrost samples, we were only able to amplify 16S rRNA gene from two samples out of four due to the low quantity of original DNA. In permafrost samples, the average number of copies was 5.96 x 10 ^5^ copies per gram of soil, with a range of 6.73 x 10 ^4^ to 2.30 x 10 ^6^ copies per gram of soil. Notably, there was considerable variation in the quantity of DNA from permafrost samples extracted with the PowerSoil Pro kit. However, the average number of 16S rRNA gene copies by soil type did not show a significant difference between the PowerSoil and PowerSoil Pro kits. Regarding garden soil samples, one sample from the PowerSoil set was lost due to technical error and dropped from further calculations. The average for the garden soil samples was 2.51 x 10 ^10^ copies per gram of soil, with a range of 3.31 x 10 ^9^ to 6.89 x 10 ^10^ copies per gram of soil. Notably, the PowerSoil Pro kit resulted in the highest number of 16S rRNA gene copies for garden soils (n = 11, Welch’s F _(2,3.34)_ = 77.22, p = .002).

To compare estimates of species richness, we used Faith's phylogenetic diversity (PD), an α-diversity metric. Faith's PD measures the sum of the minimum branch lengths of a species' phylogenetic tree, where a high number of branches indicates higher richness (Faith, 1992) . In the permafrost samples, we found that Faith's PD ranged from 21.37 to 43.10 and did not show a significant difference between the PowerSoil Pro and PowerSoil kits. Although permafrost soils showed a relatively high level of within sample diversity, they were still significantly lower than garden soil samples, which had Faith’s PD values ranging from 45.35 to 60.28 (n = 18, Welch’s t _(9.82)_ = 6.41, p < .001). We did not find a significant difference in Faith's PD values among the DNA extraction methods tested in garden soil samples. We used weighted UniFrac followed by PCoA to determine levels of similarity between sequences obtained from the three DNA extraction methods. We found that permafrost samples exhibit greater sensitivity to DNA extraction methods compared to garden soil samples. The first PCoA axis accounted for 76% of the total variation and correlated with soil type, separating garden and permafrost soils (n = 20, pseudo-F = 49.9, p = .001, q = .001). The second and third axes accounted for 15% of the variation and correlated with the DNA extraction methods. Further analysis of the permafrost samples revealed that those extracted using the PowerSoil Pro kit exhibit closer clustering than those extracted with the PowerSoil kit ( Figure 1C ). Using pairwise tests based on weighted UniFrac metrics, Permutational Multivariate Analysis of Variance (PERMANOVA) revealed a statistically significant sequence dissimilarity between permafrost samples extracted with the PowerSoil and PowerSoil Pro kits (n = 8, pseudo-F = 18.2, p = .036, q = .06). However, there were no statistically significant differences detected in sequence similarity between garden soils extracted using PowerSoil and PowerSoil Pro kits. No other statistically significant differences were found between methods for weighted UniFrac PERMANOVA pairwise analyses.

In conclusion, this study found that permafrost samples are more sensitive to differences in DNA extraction methodology compared to garden soils. Specifically, the use of the DNeasy PowerSoil Pro kit yielded significantly different results from the discontinued PowerSoil kit. These findings align with previous research indicating that differences in DNA extraction methods can affect the quantity of DNA and the phylogenetic placement of sequencing results (Whitehouse and Hottel, 2006; Vishnivetskaya et al., 2014c). The study acknowledges that the small sample size could potentially impact the generalizability of the results for some of the tested conditions. One garden soil from the PowerSoil Pro set was lost due to technical error, and two permafrost samples from the DNeasy PowerSoil Pro kit + ATL buffer set did not pass the quality filtering step during the sequencing workflow. These samples were thus dropped from ensuing analyses. These limitations should be taken into account when interpreting the conclusions of this study, which have shown that the DNeasy PowerSoil Pro kit produced the highest amount of extracted DNA and 16S rRNA gene copies per gram soil compared to other kits. Additionally, the PowerSoil Pro kit resulted in less variation in sequence similarity between permafrost samples compared to the PowerSoil kit.

This study highlights the significant impact of DNA extraction kits on the efficiency of DNA extraction, sequencing results, and interpretation of microbial community structure in permafrost samples. It emphasizes the importance of consistent DNA extraction methods in studies involving permafrost samples to ensure the accuracy and comparability of results across studies, ultimately improving our understanding of the microbial communities in permafrost and their impact on climate change.

## Methods

The following two soil types were used to evaluate the efficiency of the DNA extractions: a store-bought garden soil and a permafrost sample from Anaktuvuk, AK. The permafrost sample was collected via drilling at a depth of 95-103 cm, stored in -20°C upon collection and thawed right before use. The garden soil was stored, covered, at 25°C for one week until use.

All DNA extractions were performed using 0.250 g of soil. Each extraction was performed with four replicates and one negative control (water blank). The following methods were tested: DNeasy PowerSoil Kit (Qiagen Inc., USA), DNeasy PowerSoil Pro Kit (Qiagen Inc., USA), and a modified version of the DNeasy PowerSoil Pro Kit by adding Qiagen Buffer ATL (Qiagen Inc., USA).

The manufacturer’s protocol for the DNeasy PowerSoil kit (Cat. No. 12888-100) was followed with the following modifications: 0.250 g of soil was lysed with Solution C1 and incubated at 65°C for 5 min. The samples were then mixed at room temperature for 1 min at the 1500 speed setting using the SPEX™ SamplePrep 1600 MiniG™ (SPEX SamplePrep, Inc., Metuchen, NJ). The samples were incubated for 5 min at 4°C after the additions of Solutions C2 and C3. Final elution volume was adjusted by soil type; permafrost samples were eluted with 50 µL of Solution C6, while garden soils were eluted with 100 µL of Solution C6. All samples were incubated at room temperature for 15 min before the final centrifugation step.

The manufacturer’s protocol for the DNeasy PowerSoilPro Kit (Cat. No. 47016) was followed with the following modifications: 0.250 g of soil was lysed with 800 µL of Solution CD1 and incubated at 65°C for 5 min. The samples were then mixed at room temperature for 1 min at the 1500 speed setting using the SPEX™ SamplePrep 1600 MiniG™ (SPEX SamplePrep, Inc., Metuchen, NJ). Final elution volume was adjusted by soil type; permafrost samples were eluted with 50 µL of Solution C6, while garden soils were eluted with 100 µL of Solution C6. All samples were incubated at room temperature for 15 min before the final centrifugation step.

The protocol for the modified DNeasy PowerSoilPro kit (Cat. No. 47016) followed the procedure for the PowerSoil Pro kit with the following modification: 50 µL of ATL buffer was added to 750 µL of Solution CD1 for the lysis step.

Following extraction, DNA quantity was evaluated using the Qubit® 3.0 Fluorometer (Invitrogen, Carlsbad, CA, USA) double-stranded DNA (dsDNA) high-sensitivity (HS) assay. The manufacturer’s protocol was used with the following modifications based on soil type: The quantity of DNA in the permafrost soils was determined using 20 µL of sample, while 1 µL of sample was used for garden soils. To account for the difference in sample volume, DNA quantity calculations were adjusted accordingly in the Qubit® 3.0 Fluorometer. All DNA extractions were stored at -20°C until further analysis.

Following extraction and quantification, a qPCR assay targeting the 16S rRNA gene in the V3-V4 region and library preparation was performed using the Zymo Research Quick-16S™ Plus NGS Library Prep Kit (V3-V4) (Zymo Research, Irvine, CA, USA) according to the manufacturer’s instructions with the following modifications: The PCR master mix was prepared with 10 µL of Equalase qPCR Premix and 2 µL of DNase/RNase-free water per sample. Each reaction mixture consisted of 12 µL of master mix, 2 µL each of i5 and i7 primers from the V3-V4 Index Primer Set 5, and 4 µL of the DNA sample. DNA samples were diluted so that the input did not exceed 100 ng. Garden soils extracted with PowerSoil Pro were diluted 5X, while garden soils extracted with PowerSoil Pro + ATL were diluted 2X. One garden soil sample from the PowerSoil Pro set was lost due to technical error. Standards were prepared in triplicate with a 10-fold dilution series from 10 ^6^ to 10 ^1^ copies per µL of the kit-provided ZymoBIOMICS™ 16S/ITS qPCR Standard. The positive control was prepared using the kit-provided ZymoBIOMICS™ Microbial Community DNA Standard (Zymo Research, Irvine, CA, USA), which was diluted 2X for a starting concentration of 25 ng/µL. The negative control was prepared with DNase/RNase-free water. Thermal cycling conditions were 1 cycle at 95°C for 10 min and 35 cycles of the following three steps: 95°C for 30 s, 55°C for 30 s, and 72°C for 180 s. All samples were held at 4°C following amplification. The optimization values were as follows: efficiency = 0.986, r = 0.997, and r ^2^ = 0.994.

Following PCR amplification, 12 µL of each permafrost sample and 5 uL of each garden soil sample were combined and 50 µL of the provided PCR Inactivator Solution was added to make a total volume of 225 µL. The final library clean up was executed according to the manufacturer’s instructions with the following modification: the final elution was done with 30 µL of Tris-HCl solution (10mM, pH = 7.5). The final Qubit HS dsDNA value was 41.1 ng/µL. Final libraries were analyzed for size using a Bioanalyzer High Sensitivity kit (Agilent, Santa Clara, CA, USA). The pooled 16S amplicon libraries were sequenced on a single lane using MiSeq v2 NANO 150 PE at UC Berkeley's California Institute for Quantitative Biosciences.

All blank extraction controls were checked with PCR using 338F and 515R primers with 35 PCR cycles and gel electrophoresis to confirm no amplification. Each PCR reaction had a total volume of 25 µL and consisted of 2.5 μL Takara ExTaq (TaKaRa Corp., Shiga, Japan) 10X buffer, 2μL dNTP mix (2.5 mM), 0.7 μL Roche BSA (20 mg/mL), 1 μL each of forward and reverse primers (400 nM final concentration), 0.25 μL Takara HotStart Ex Taq DNA Polymerase (5U/μl), 16.55 μL PCR-grade water, and 1 μL of sample. Thermal cycling conditions included an initial denaturation step at 98°C for 3 min, followed by 35 cycles of 98°C for 30 s, 52°C for 40 s, 72°C for 90 s, and a final extension at 72°C for 5 min.

Following library preparation, sequencing analysis was done in Quantitative Insights into Microbial Ecology (QIIME) 2 following DADA2 (Callahan et al., 2016) . Raw reads went through a quality filtering to remove low quality (<q30) reads. Two permafrost samples that were extracted with the DNeasy PowerSoil Pro kit with Qiagen ATL buffer did not pass this initial quality filtering due to the low quantity of reads and were excluded from further analysis. Based on an interactive quality plot generated by the demultiplexing summary in the last step, DADA2 was used to trim off the first 6 bases and truncate sequences at position 150. Sequences were then grouped into operational taxonomic units (OTUs) based on 99% sequence similarity, and representatives were chosen from each set with a minimum sampling set of 12,533 sequences per sample. Singletons, mitochondria, and chloroplasts were removed and communities were standardized to a total number of 12,553 sequences per sample. Sequences were aligned with Multiple Alignment using Fast Fourier Transform (MAFFT), and FastTree was used to create a phylogenetic tree that was used for the calculation of α- (Faith’s PD) and β-diversity (weighted UniFrac distance) metrics. The weighted UniFrac distance matrices were used to visualize sequence similarity among extraction kits using principal coordinate analysis. Sequencing reads are deposited in the NCBI Sequence Read Archive (SRA) under BioProject PRJNA954778.

**Statistical Analysis**

Significant differences between data sets were identified by Welch's analysis of variance (ANOVA) as implemented in the ggstatsplot (Patil, 2021) package using R v. 4.0.2 (R Development Core Team, 2008) . P-values are corrected for multiple comparisons via the Holm–Bonferroni method. PCoA and PERMANOVA analyses were performed with QIIME 2 (Bolyen et al., 2019) . Graphs were assembled into the figure with the GIMP 2.10.22 GNU Image Manipulation Program.

## Reagents

**Table:** 

Kit/Reagent	Cat. No. /ID	Use
Qiagen DNeasy PowerSoil Kit Protocol	12888-100	DNA Extraction
Qiagen DNeasy PowerSoil Pro Kit	47016	DNA Extraction
Zymo Research Quick-16S™ Plus NGS Library Prep Kit (V3-V4) with Primer Set 5	D6422	16S Library Prep
Qiagen Buffer ATL	939011	DNA Extraction

**Table:** 

Primer	Sequence (5' to 3')	Use
341f	A mixture of CCTACGGGDGGCWGCAG and CCTAYGGGGYGCWGCAG	16S Library Prep (qPCR)
806r	GACTACNVGGGTMTCTAATCC	16S Library Prep (qPCR)
338f	ACTCCTRCGGGAGGCAGCAG	Standard PCR
515r	CCTACGGGAGGCAGCAG	Standard PCR
